# Conformational Polymorphism of m^7^GTP in Crystal Structure of the PB2 Middle Domain from Human Influenza A Virus

**DOI:** 10.1371/journal.pone.0082020

**Published:** 2013-11-29

**Authors:** Toshiharu Tsurumura, Hao Qiu, Toru Yoshida, Yayoi Tsumori, Dai Hatakeyama, Takashi Kuzuhara, Hideaki Tsuge

**Affiliations:** 1 Faculty of Life Sciences, Kyoto Sangyo University, Kamigamo-Motoyama, Kyoto, Japan; 2 Faculty of Pharmaceutical Sciences, Tokushima Bunri University, Tokushima, Japan; The Scripps Research Institute, United States of America

## Abstract

Influenza pandemics with human-to-human transmission of the virus are of great public concern. It is now recognized that a number of factors are necessary for human transmission and virulence, including several key mutations within the PB2 subunit of RNA-dependent RNA polymerase. The structure of the middle domain in PB2 has been revealed with or without m^7^GTP, thus the middle domain is considered to be novel target for structure-based drug design. Here we report the crystal structure of the middle domain of H1N1 PB2 with or without m^7^GTP at 1.9Å and 2.0Å resolution, respectively, which has two mutations (P453H, I471T) to increase electrostatic potential and solubility. Here we report the m^7^GTP has unique conformation differ from the reported structure. 7-methyl-guanine is fixed in the pocket, but particularly significant change is seen in ribose and triphosphate region: the buried 7-methyl-guanine indeed binds in the pocket forming by H357, F404, E361 and K376 but the triphosphate continues directly to the outer domain. The presented conformation of m^7^GTP may be a clue for the anti-influenza drug-design.

## Introduction

Influenza virus is an RNA virus belonging to the *Orthomyxoviridae* family, characterized by eight segments of viral RNA. In humans, seasonal influenza virus causes respiratory inflammation, high fever, head and muscle aches, and fatigue. However, during the 1918 pandemic, the influenza virus (known as the Spanish influenza) also caused severe pneumonia, and an estimated 50 million died worldwide [1]. Including that event, there have been four pandemics: the Spanish influenza (H1N1) in 1918/1919, Asian influenza (H2N2) in 1957, Hong Kong influenza (H3N2) in 1968 and H1N1 influenza in 2009. Within the influenza virus, three RNA-polymerase proteins (PA, PB1 and PB2), the nucleoprotein (NP) and the viral RNAs form the ribonucleoprotein (RNP) complex [2]. The polymerases within this complex catalyze the transcription and replication of the viral genome, while NP forms an oligomerization complex to stabilize the vRNA. Among the polymerases, PB1 plays the central role in RNA polymerization. PB2 mediates the cap-snatching mechanism; i.e., it binds the capped mRNA of the host for transcription of the influenza genome. And PA acts as a nuclease, cleaving the capped mRNAs to produce 13- to 15-mers, which serve to prime viral mRNA transcription [3].

The structure of the RNP complex were investigated using cryogenic electron microscopy [4,5], however, there have been no structural analyses of the entire RNP complex or the RNA polymerase complex at the atomic level. Partial domain structures of the RNA polymerase subunit have been reported [6-11]. In PB2, substitutions at positions 627 of PB2 have been reported to be important for the adaptation of the avian virus to mammalian hosts [12-14]. The crystal structures of the large C-terminal domain of PB2, including this E627K mutation, which enables human infection, were reported by us and other [6,15]. Our structure is the first deposited structure of a PB2 domain containing the pathogenicity determinant lysine 627 in the Protein Data Bank (2008 Apr 21) and we also revealed the RNA binding ability of this domain which is strengthened by the E627K mutation [15]. On the other hand, though several potential cap binding sites had been postulated based on crosslinking and mutagenesis experiments: 242-252 as N-site [16], 533-577 as C-site [16,17] and a more central site as M-site [18], the complex structural analysis with m^7^GTP showed that the cap-binding sites is the middle domain of PB2 [19]. Recently further structures of the PB2 middle domain from three strains were reported; human H1N1 middle domain without m^7^GTP, human H3N2 middle domain with m^7^GTP and human H5N1 middle domain with m^7^GTP [20].

**Figure 1:**
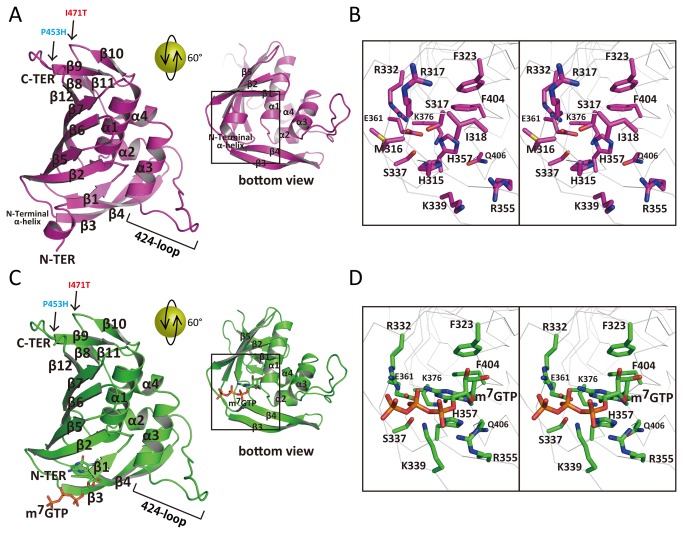
Crystal structure of PB2 middle domain (amino acids 318 to 483) of H1N1 from human influenza A virus with or without m^7^GTP. A) Crystal structure without m^7^GTP in magenta. Left panel is the overall structure. Right panel is the same model as left panel but rotated by 60° about a horizontal axis to show the active site. Secondary structures are labeled in black and two mutations P453H and I471T are indicated by arrows with blue and red labels, respectively. B) Close-up view of the square of panel A in stereo view. Main chain is represented by white ribbon. Residues of active site and N-terminal helix are labeled in black. C) Crystal structure with m^7^GTP in green. Left panel is the overall structure. Right panel is the same model as left panel but rotated by 60° about a horizontal axis to show the active site. m^7^GTP is represented by stick model. Secondary structures are labeled in black and two mutations P453H and I471T are indicated by arrows with blue and red labels, respectively. D) Close-up view of the square of panel of C in stereo view. Main chain is represented by white ribbon. Active site residues are labeled in black. H357, F404, E361 and K376 formed the active site cleft to bind to 7-methyl-guanine. Triphosphate interacts with R332, S337, K339, R355 and E361.

Here we report PB2 middle domain structure (318-483) belonging to subtype H1N1 human influenza with m^7^GTP (Figure 1), which has two mutations (P453H, I471T) to increase solubility of this domain. Though the whole structure of PB2 middle domain is basically the similar as the reported structure, the appeared conformation of m^7^GTP is different from the reported one. We discuss about the m^7^GTP conformation which would be important for the drug design.

## Results

### Properties of the middle domain of PB2 (H1N1)

We initially purified His-tagged PB2 middle domain using a nickel chelate column [19]. However, the fraction eluted with 200 mM NaCl was not stable, and aggregation of the protein could be seen. We therefore modified the protocol to elute the protein with 500 mM NaCl and found that the PB2 (H1N1) middle domain was stable under these high salt conditions. Next we considered to change the surface electrostatic potential and to increase the middle domain solubility by mutations. Two mutations (P453H and I471T) were introduced because these positions are surface of the protein and far from cap-binding site. These mutations increased the solubility so that the final protein was stable in 50 mM Tris-HCl (pH 8.0), 200 mM NaCl. Crystals appeared in two or three days and reached to ~0.3mm size. We collected the native data sets at 2.00 Å resolution with in-house X-ray generator and detector (Table 1). We also collected the data set of m^7^GTP soaked crystal in 0.5 mM m^7^GTP for 2.5 hours, at 1.93 Å (Table 1). The upper limit resolution of these data were carefully chosen including the recent discussion of CC_1/2_ [21]. The final *R*work (*R*free) of apo and m^7^GTP-bound form were 18.6% (22.8%) and 17.8% (21.2%), respectively.

**Table 1:** Data collection and structure refinement statistics of PB2 middle domain.

Data collection			
	Apo form	m^7^GTP-bound form	
Unit cell (Å)	a=107.8, b=107.8, c=136.6	a=107.6, b=107.6, c=138.3	
Space group	R32	R32	
Beamline	Micro7HFM *RAXISVII*	Micro7HFM *RAXISVII*	
Resolution (Å)	20.98-2.00 (2.07-2.00)	18.88-1.93 (2.00-1.93)	
Total number of reflections	225,288(20,870)	166,528 (15,742)	
Number of unique reflections	20,909 (2,041)	23,160 (2,272)	
Completeness (%)	99.8 (98.3)	99.06 (99.43)	
*R*merge	0.104 (1.055)	0.058 (0.622)	
*R*meas	0.109 (1.110)	0.063 (0.673)	
*R*pim	0.033 (0.346)	0.023 (0.253)	
I/sigma(I)	24.8 (3.2)	29.3 (4.4)	
Redundancy	10.8 (10.2)	7.19 (6.93)	
CC_1/2_	0.999 (0.698)	0.999 (0.782)	
Refinement			
*R*work	0.186		0.178
*R*free	0.228		0.212
RMSD bond length	0.007		0.006
RMSD bond angle	1.032		1.076
Number of atoms in the model	1,529		1,555
Number of water molecules	185		233
Number of residues in outlier region of Ramachandran plot	0		0
The average B-factor	33.5		34.2

Values in parentheses are for the last resolution shell.

*R*merge = ∑*hkl*∑*i*|*li*(*hkl*) - <*l*(*hkl*)>|*l*∑*hkl*∑*i*|*li*(*hkl*)|

*R*meas = ∑*hkl*[N/(N-1)]^1/2^∑*i*|*li*(*hkl*)-<*l*(*hkl*)>|*l*∑*hkl*∑*i li*(*hkl*)

*R*pim = ∑*hkl*[1/(N-1)]^1/2^∑*i*|*li*(*hkl*)-<*l*(*hkl*)>|*l*∑*hkl*∑*i li*(*hkl*)

where *li*(hkl) is the intensity measurement for a reflection *hkl*,

<*l*(*hkl*)> is the mean intensity for this reflection and N is Redundancy.

CC_1/2_ is calculated according to the formula in Ref. 21.

*R*work= ∑*hkl*||*Fobs*| - |*Fcalc*||*l*∑*hkl*|*Fobs*|.

*R*free was calculated with randomly selected reflections (5%).

### Structure of the H1N1 PB2 Middle Domain

**Figure 2:**
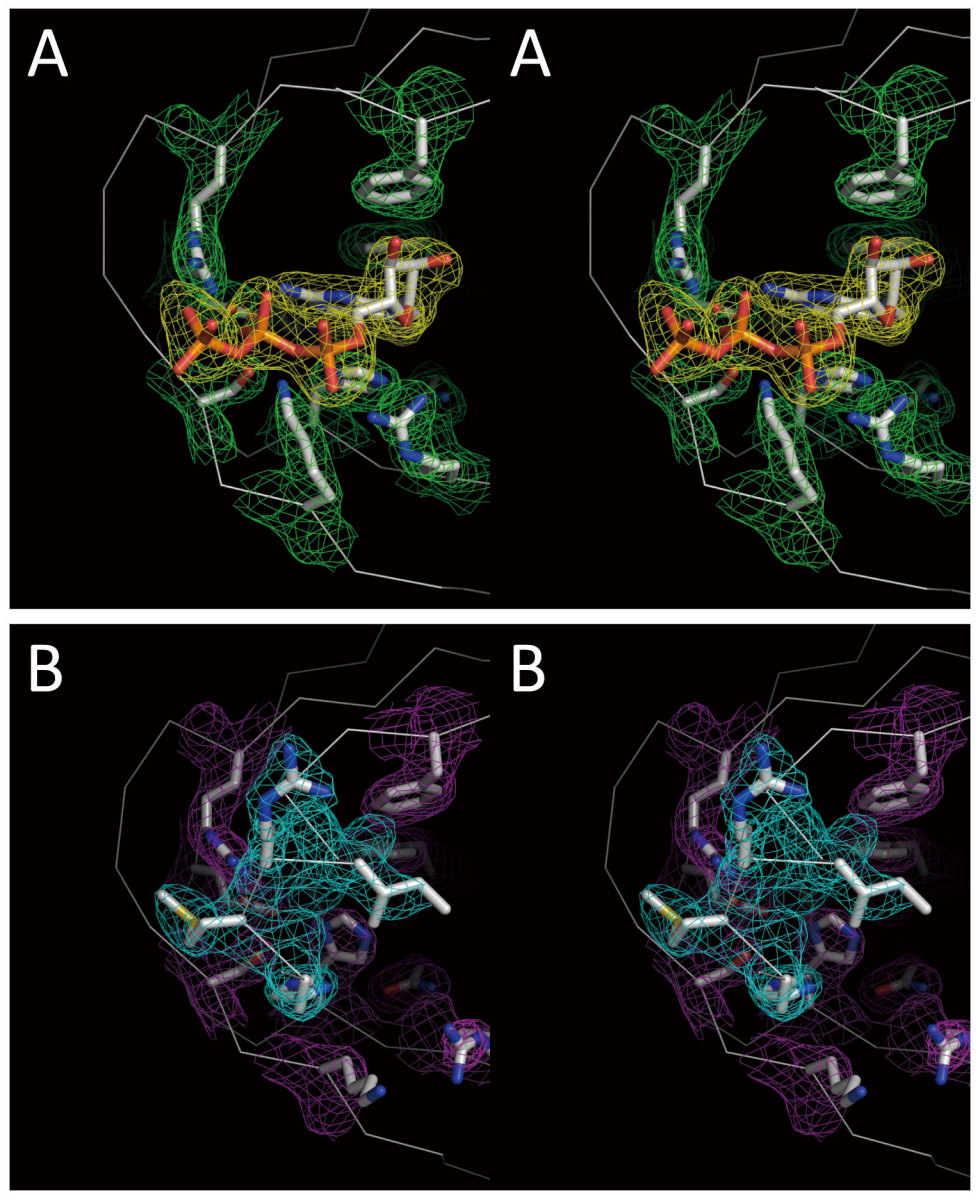
Electron densities of the active sites in stereo view. The molecular orientation is the same as bottom view in Figure 1. The 2Fo-Fc electron density maps are drawn at one sigma. A) The active site of m^7^GTP-bound form. The electron density of m^7^GTP and the active site residues are represented in yellow mesh and green mesh, respectively. 6B) The active site without m^7^GTP. The electron density of additional N-terminal helix and the active site residues are represented in cyan mesh and magenta mesh, respectively.

There was one molecule in an asymmetric unit (Figure 1). This crystal contains large solvent content (70%), and two mutations P453H and I471T located on the solvent accessible surface in the crystal packing. The similar minimal cap binding domain structure has been revealed before (2VQZ). The overall fold was the same as reported structure: a four helix bundle (α1 ~ α4) was wrapped by three parts of β-sheet ((1) β1, β2, β5, β6 and β7 (2), β8 〜 β12 and (3) β3 ~ β4). However, compared with the reported structure of PB2 middle domain, in native, one large structural change could be seen: N-terminal region formed one additional α-helix which consists of ^315^SHMRISS^321^ (Figure 1 and Figure 2). The first three residues (^315^SHM^317^) are additional residues derived from pET15b vector. The side chain of H316 interacts with S336 and it seems to stabilize this α-helix. On the other hand, by m^7^GTP soaking, the α-helix deformed to a flexible structure as describe later. The RMSD value is 1.00 Å between native and m^7^GTP using all identical atoms. A homology structural search using DALI [22] showed no strong similarity with any other proteins except 2VQZ. This is also true for the PB2 large C-terminal domain, which shows no strong similarity to any other proteins. Apparently influenza A virus PB2 is very distant from other proteins, at least based on its structural classification.

### Conformational Polymorphism of m^7^GTP

**Figure 3:**
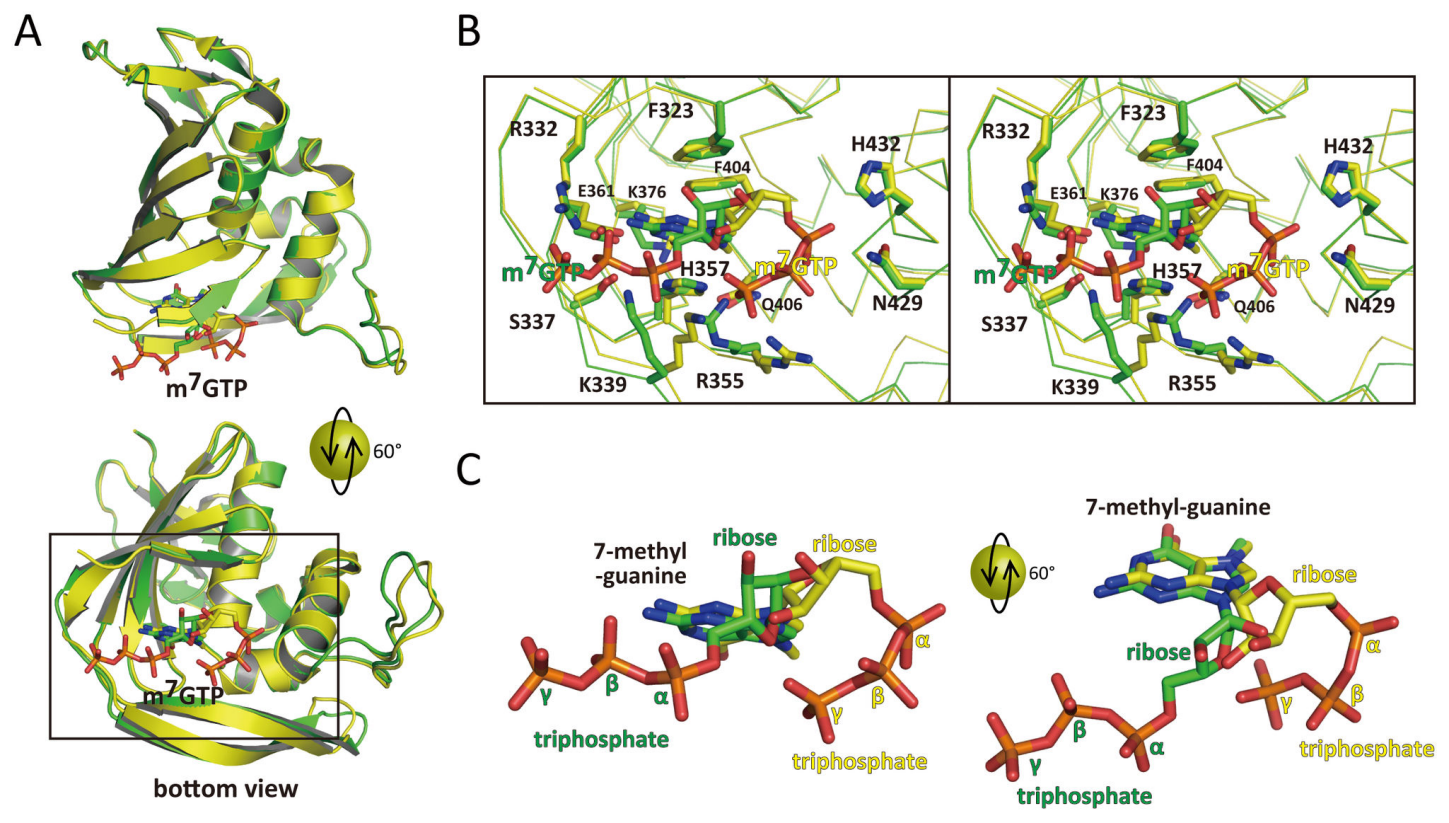
Comparison the m^7^GTP-bound form in H1N1 (this report) and H3N2 (2VQZ). A) Crystal structure in H1N1 and H3N2 are shown in green and yellow, respectively. Both m^7^GTPs are depicted with stick model. Upper panel is the overall structure and lower panel is the bottom view with 60° orientation of upper panel. B) Close-up view of the square of panel A in stereo view. Main chain is represented by ribbon. Active site residues are labeled in black. C) Comparison the conformation of m^7^GTP in H1N1 and H3N2 structure. Left panel is same orientation as A and B. Right panel is the same model as left panel but rotated by 60° about a horizontal axis to show the active site.. The position of ribose and triphosphate are labeled in each color. The 7-methyl guanine is same conformation and it is labeled in black.

The crystal was soaked in 0.5 mM m^7^GTP solution for 2.5 hours. The 2Fo-Fc electron density of m^7^GTP was explicitly visible (Figure 2). Compared with the reported structure (Figure 3), it shows the similar conformation that 7-methyl-guanine is fixed in the deep cleft forming by H357, F404, E361 and K376. However, particularly significant change was seen in ribose and triphosphate region. In reported structure (2VQZ), the triphosphate is bent around the base with the α-phosphate interacting with H432 and N429 and the γ-phosphate interacting with H357 and, K339 and R355 (Figure 3). In our structure, such bent could not be seen because the ribose rotated via N9 and C1 so the triphosphate continues directly to the outer domain (Figure 1C, D left direction). Interestingly, compared with native structure, the N-terminal α-helix deformed to a flexible structure upon the soaking of m^7^GTP, thus the triphosphate lies in the same region instead of α-helix. It is important to note that the N-terminal region is flexible with no electron density, so the conformation m^7^GTP is not affected by these N-terminal residues. Furthermore, in two more structures of PB2 middle domain with m^7^GTP, all m^7^GTP conformation is the same as that in 2VQZ [19]. Conformational features of the presented m^7^GTP are listed as follows: (1) the side chain of R355 and K339 changed the direction to hold the triphosphate. The feature is not seen in other PB2 middle domain structures with m^7^GTP (2). The side chain of H357 moves from apo to holo state to interact with 7-methyl-guanine via π-π interaction (3). The conformation of m^7^GTP in 2VQZ shows the interaction with N429, M431 and H432, but the presented structure shows no interaction with these residues.

## Discussion

### P453H mutation increased the surface basic electrostatic potential drastically

**Figure 4:**
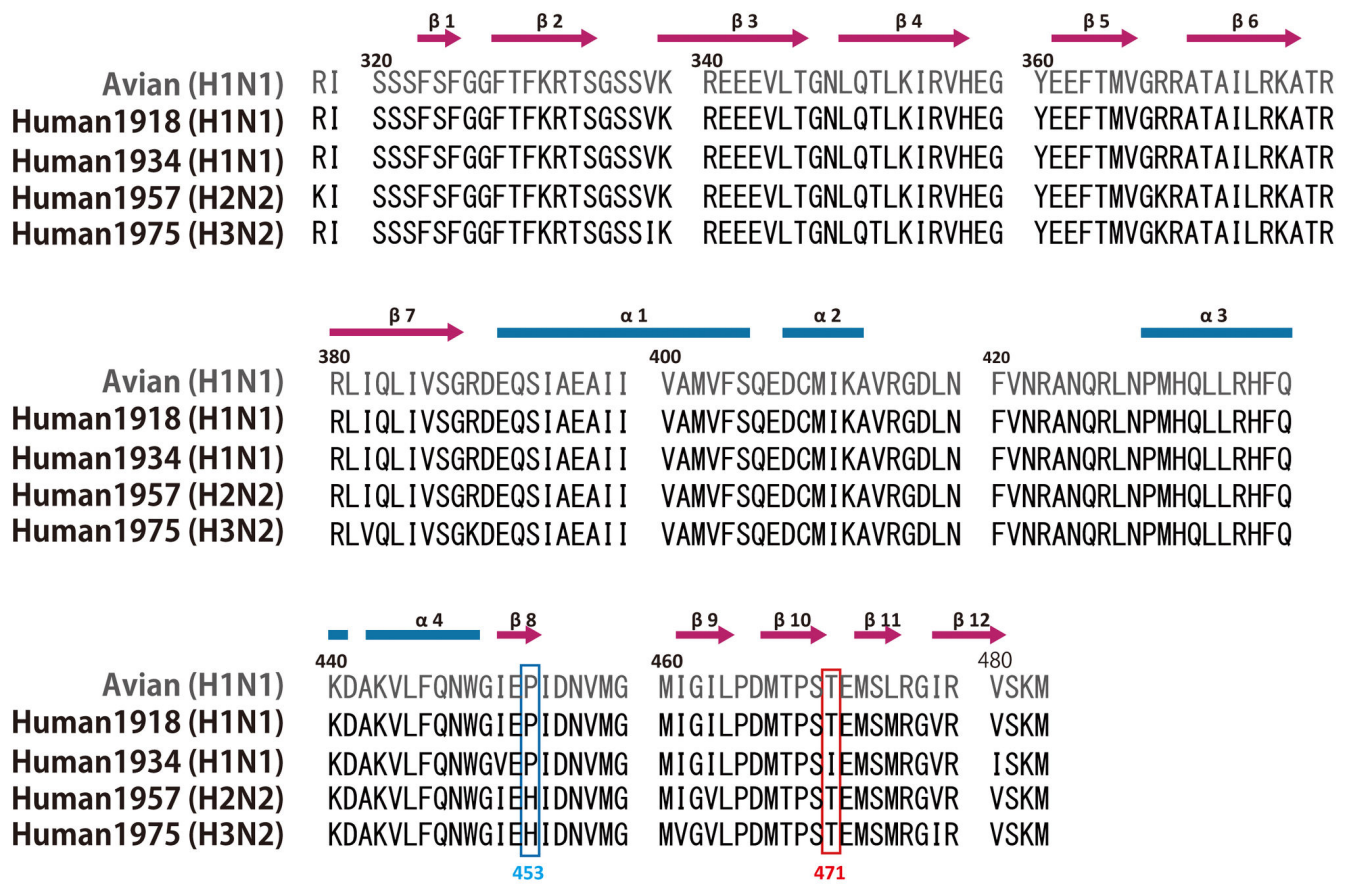
Sequence alignment of the PB2 middle domains. Avian strain is shown in grey (A/mallard/Tennessee/11464/1985 (H1N1)). Human influenza virus is shown in black: H1N1 (A/Brevig Mission/1/1918), H1N1 (A/Puerto Rico/8/1934), H2N2 (A/Albany/20/1957), and H3N2 (A/Victoria/3/1975) as 2VQZ. The blue and red boxes are the mutation sites at 453 and 471 (See details in the text). Twelve β-strands and four α-helices are depicted in hot pink arrows and light blue bar, respectively.

In Figure 4, we showed the sequence differences of PB2 middle domain of avian influenza H1N1 and human influenza H1N1, H2N2 and H3N2. In the whole PB2 sequence, it was reported that seventeen substitutions, which are highly conserved in avian or human, would be candidates to enable to cross the barrier between the species from avian to human as an adaptive mutant [23]. In addition to the seventeen characteristic sites, they reported that two more additional characteristic sites unique to the H1N1 (human), and nine unique to the HxN3 (H2N2/H1N2/H3N2, human). These eleven variants would be also candidates which are needed to cross the species barrier between avian H1N1 and human HxN2. There are no overlap between these sites and the former seventeen mutations [23]. Within former seventeen substitutions, seven sites at position 567, 588, 613, 627, 661, 674, and 702 are highly conserved in the C-terminal domain of PB2. Especially, at 627 position, glutamate is conserved in avian H1N1 with 99.31% and lysine is conserved in human H1N1 and HxN2 with 99.76% [23]. E627K mutation dramatically increased basic charge on the surface. It facilitated the adaptation of H5N1 and other avian viruses to mammals and increased their transmission and/or pathogenicity in humans, mice, ferrets and guinea pigs [24]. Within the PB2 middle domain, there are each two sites with the change from avian to human (residues 368 and 475 (1^st^ human isolate in 1940 and 1918, respectively)) and from avian to H2N2/H3N2 (residues 382 and 453 (1^st^ human isolate in 1961 and 1940, respectively)) [23]. These sites are not in the vicinity of the cap-binding site. Among these four sites, the mutation at position 453 is particularly interesting because it is so drastic, going from proline (H1N1) to histidine (H3N2), though other mutations occur within similar character such as basic (R368K) or hydrophobic (I382V). The P453H mutation first appeared in 1940. Residue 453 is Pro (or Ser) with 98.29% conservation in avian H1N1, and His is conserved in human HxN2 with 99.49%. Thus, based on the characteristic features of P453H mutation, it might be more possible candidate than the others for an adaptive mutation from avian to human HxN2 [23]. In this report, we observed that the PB2 middle domain with two mutations (P453H and I471T) change the solubility and finally we obtained good diffraction crystals by these mutations. It has been well known that the electrostatic surface change by the single E627K mutation enhanced the virulence [15], though it is still open question what is the role of K627 in the RNP complex. Whether the P453H mutation is linked to the viral specific feature of H2N2 or H3N2 is an important issue to be addressed. Given that the histidine at 453 is present in H2N2 or H3N2 but not H1N1, this site may contact with other components such as RNA, PB2, PB1, PA, NP and nuclear export protein [25].

### Importance of m^7^GTP Conformation in the Trimeric RNA-Polymerase Complex

The novel conformation of m^7^GTP we presented will be important clue for drug design targeting the cap-binding site. It should be noticed again that two mutations (P453H and I471T) we introduced are totally opposite surface against cap-binding site, thus they does not influence the conformational polymorphism of m^7^GTP (original (2VQZ) and novel site (this report)). 

**Figure 5:**
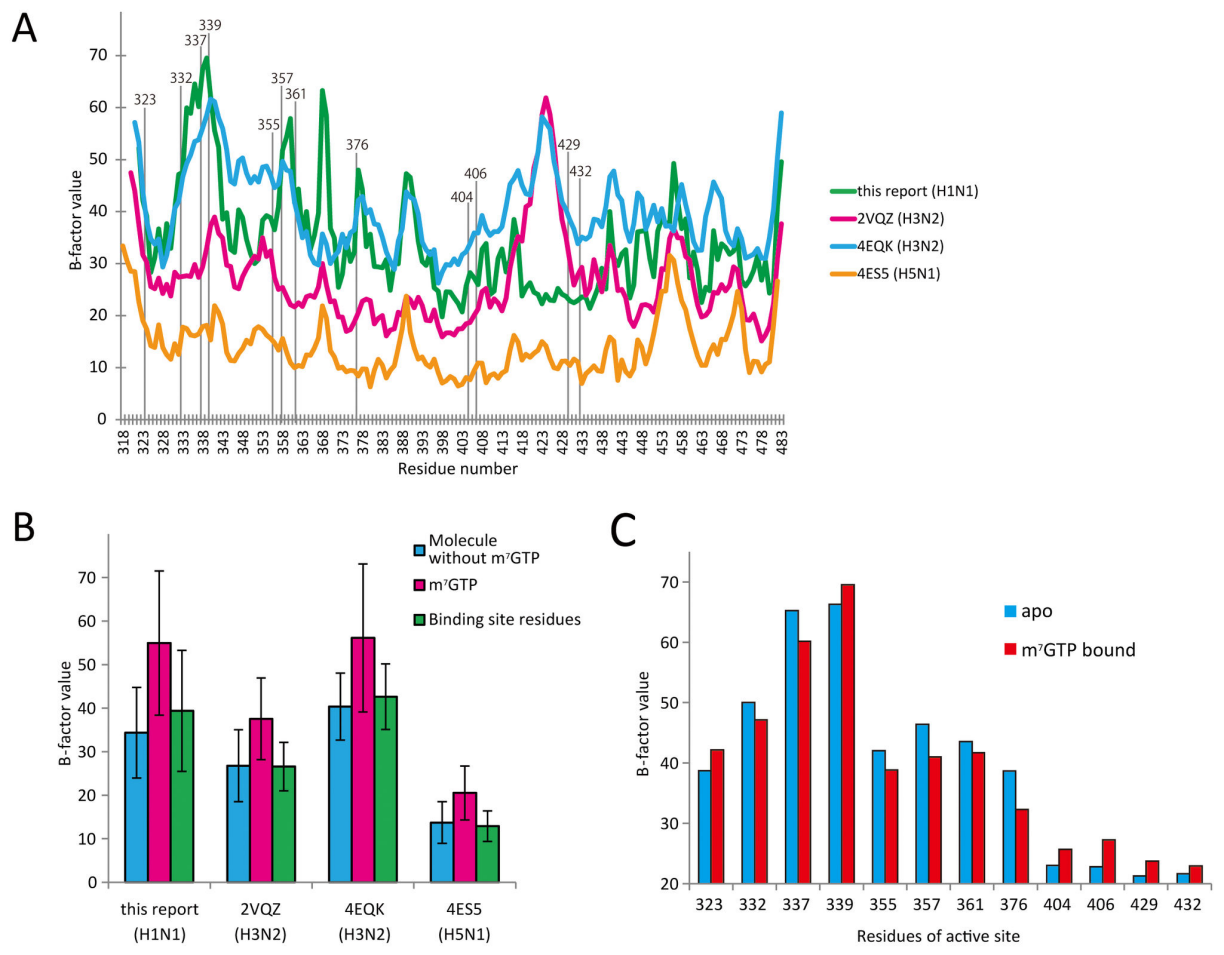
B-factor plot for Cα carbons in m^7^GTP-bound structures. A) B-factor of the structure in this report is plotted in green, 2VQZ is in magenta, 4EQK is in cyan and 4ES5 is in orange. Active site residues are indicated by vertical lines and labeled in black. B) Mean B-factor values of whole molecule without m^7^GTP (cyan), m^7^GTP only (magenta) and active site residues bound to m^7^GTP (green). C) Changes of B-factor values of the active site residues between apo (blue) and m^7^GTP (red).

As we applied m^7^GTP by crystal soaking, the conformation of soaked compound may be restricted by crystal packing. Thus we checked next points about the phosphate binding site: (1) Original site was preserved in our crystal statically: 7-methyl guanine occupies the same site, but phosphate binds in different manner. There is no direct disturbance to bind phosphate in original site in our crystal. In the original site, important residues for the phosphate binding (H432 and N429) did not change the position (2). Original site was preserved in our crystal dynamically: In comparison with B-factor plot of four different PB2 structure with m^7^GTP, there are two regions (338 region and 424 loop region) with large difference (Figure 5A). It is considered that these regions are candidate which are influenced by crystal packing. In the same figure, the binding residues were plotted. It shows the phosphate binding site (H432 and N429) are not influenced because they are outside the 338 and 424-loop region. Furthermore, though the phosphate binds R332, S337 and K339 on the 338 region in our structure, we checked that the 338 region of our crystal is not restricted by molecular packing. Based on these facts, we concluded that the novel site is not artifact by the soaking and crystal packing.

In our crystal, there was less interaction between the molecules because solvent content is 70%. On the other hand, in other crystals (2VQZ and E4S5), the solvent content are ~54%. Average B-factor of our crystal might be the relatively high B-factor compared with 4ES5 (H5N1), but is almost similar compared with 4EQK (H3N2) (Figure 5B). This is the first report with or without m^7^GTP using the same crystal form. B-factors of some residues around m^7^GTP (S332, S337, R355, H357, E361 and K376) are suppressed upon m^7^GTP binding (Figure 5C). 

**Figure 6:**
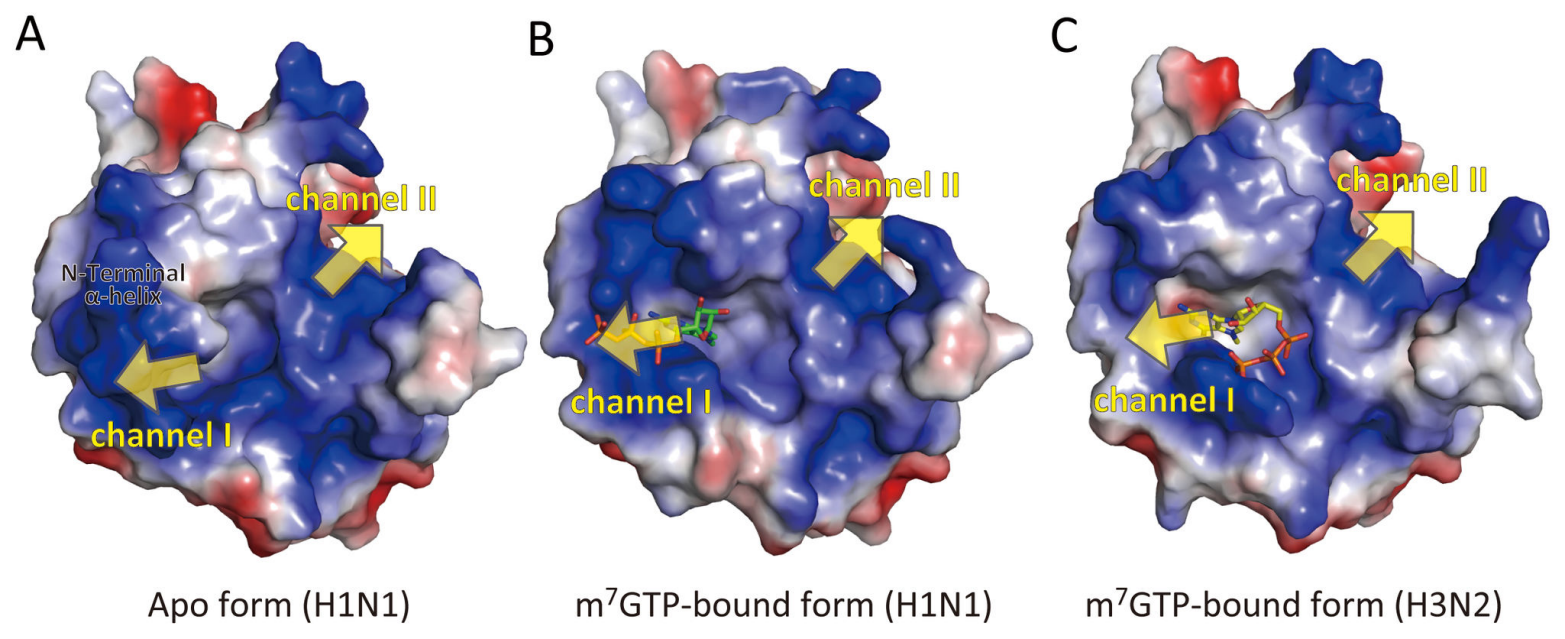
Electrostatic surfaces of the PB2 middle domain. A) Apo form and B) m^7^GTP-bound form in H1N1 and C) m^7^GTP-bound form in H3N2. The molecular orientation is the same as bottom view in Figure 1 and Figure 3. Yellow arrows show the channels, which might bind the continuous RNA. Electrostatic surfaces were calculated and displayed using PyMOL.

In the first PB2 middle domain structural paper, it suggested the possibility of the different conformation of triphosphate in the context of a capped oligonucleotide. In electrostatic surface of the cap-binding site (Figure 6), we could see channels with strong basic charge in two regions. One region includes R335, K339, K331 and R332 (channel I) and the other region includes R423 on the 424-loop, R436 and R368 (channel II). We observed that the capped-oligonucleotide interacts with channel I in the conformation, but we don’t deny the possibility the continuous RNA interacts with channel II in the trimeric RNA-polymerase complex (Figure 6). We still don’t know which way the capped-oligonucleotide goes through in the trimeric RNA-polymerase complex and this will be next important issue to be addressed.

## Materials and Methods

### Gene cloning, expression and protein purification

The *pb2* gene (RIKEN) was inferred from the amino acid sequence of influenza virus H1N1 (A/Puerto Rico/8/1934). We used DNA encoding residues 318-483 subcloned into pET15b vector between the NdeI and BamHI sites. The PB2 middle domain (318-483) was then expressed with an N-terminal histidine-tag and a thrombin cleavage site in *E. coli* BL21-CoDonPlus-RIL cells (Agilent Technologies) growing in LB medium. The protein was purified by affinity capture on a Ni-IDA 2000 column (MACHEREY - NAGEL). After elution with imidazole, the protein was cleaved with thrombin and loaded onto an Uno S column (Bio-Rad) and eluted with a salt gradient (0.5 M - 1 M NaCl). The eluted PB2 middle domain was not dissolved well under 500 mM NaCl concentration. To obtain more soluble domain, we introduced two mutations on the surface of this domain: P453H and I471T. The final protein was then concentrated to 10 mg/ml in 50 mM Tris-HCl (pH 8.0), 200 mM NaCl and 2 mM DTT and stored at -80°C. At N-terminal and C-terminal, there are additional four residues each ^314^,GSHM^317^ and ^481^GSGC^484^, respectively.

### Crystallization

Crystals were grown using the hanging-drop vapor diffusion method. The protein solution was mixed with an equal volume of well solution (1.2 M NaCl and 2.5% (v/v) ethanol) and incubated at 4°C.

### Data collection and structure determination

Crystals were picked up using a nylon loop. They were then dipped in mother liquor containing 20% (v/v) ethyleneglycol as a cryoprotectant and plunged into a nitrogen-gas stream at 100 K. The data were collected using an X-ray wavelength of 1.54 Å using Micro7HFM *RAXISVII*. The diffraction images were indexed, integrated and scaled using HKL2000 program [26]. The crystal space group was determined to be *R32*. Initial phase was obtained by molecular replacement (phaser) using H3N2 cap-binding domain structure 2VQZ [27]. The structure was refined using phenix.refine restraint refinement and TLS refinement [28]. Model building and manual refinement was done by Coot [29]. Comparison of the structures was done using PyMOL [30]. Data statistics such as *R*merge, *R*meas, *R*pim and CC_1/2_ were obtained by Xtriage of phenix [31].

The missing residues of electron density are G314 (N-terminal) and ^481^GSGC^484^ (C-terminal) in native structure and ^314^GSHMRS^319^ (N-terminal) and ^481^GSGC^484^ (C-terminal) in m^7^GTP structure, respectively.

### Accession Numbers

Coordinates and structure factors of PB2 middle domain with two amino acids mutation (P453H and I471T) have been deposited in the Protein Data Bank. The accession numbers of the structure without m^7^GTP and with m^7^GTP are 3WI0 and 3WI1, respectively. Additionally, wild type of PB2 middle domain without m^7^GTP has been deposited with the accession number 4J2R (This is not mentioned in this manuscript, but it is related structure of this manuscript.).
